# Implications of Long Sleep Duration on Cardiovascular Health: A Systematic Review

**DOI:** 10.7759/cureus.77738

**Published:** 2025-01-20

**Authors:** Christian Sanchez Corredera, Pranav S Tadepalli, Julian Scaccia, Adiraj S Sibia, Harvey N Mayrovitz

**Affiliations:** 1 Medicine, Nova Southeastern University Dr. Kiran C. Patel College of Osteopathic Medicine, Davie, USA; 2 Cardiothoracic Surgery, Nova Southeastern University Dr. Kiran C. Patel College of Osteopathic Medicine, Davie, USA; 3 Medical Education and Simulation, Nova Southeastern University Dr. Kiran C. Patel College of Allopathic Medicine, Davie, USA

**Keywords:** atherosclerosis, cardiovascular disease, cardiovascular health, coronary artery disease, heart failure, hypertension, inflammatory markers, long sleep duration, short sleep duration, u-shaped association

## Abstract

Sleep duration is an important determinant of cardiovascular health, yet the adverse effects of long sleep duration remain underexplored. While short sleep has well-documented associations with increased cardiovascular risk, emerging evidence highlights a U-shaped relationship, with excessive sleep also linked to adverse outcomes. This systematic review evaluates the association between prolonged sleep duration and cardiovascular health outcomes, including coronary artery disease (CAD), stroke, myocardial infarction (MI), hypertension, heart failure (HF), and atherosclerosis. A systematic search of PubMed, Embase, and Web of Science identified 38 studies published between 2008 and 2024 that investigated the relationship between sleep duration and cardiovascular health. Prolonged sleep duration was consistently associated with increased risks of CAD, stroke (ischemic and hemorrhagic), hypertension, and MI. Elevated inflammatory markers, such as C-reactive protein (CRP) and interleukin-6 (IL-6), emerged as potential mediators alongside demographic and lifestyle factors, including age, gender, and socioeconomic status. Long sleep duration may serve as a modifiable risk factor for cardiovascular diseases. Incorporating sleep assessments into cardiovascular risk evaluations could inform prevention strategies, and further research is needed to elucidate mechanisms and develop targeted interventions.

## Introduction and background

Sleep duration, a crucial component of daily life, has garnered considerable attention in the field of public health and medicine because of its profound implications for overall well-being. While the detrimental effects of short sleep duration on cardiovascular health have been extensively documented and widely disseminated - such as associations with increased risks of hypertension, coronary artery disease (CAD), and stroke [1] - the lesser-known repercussions of long sleep duration have often been overlooked. The optimal sleep duration required for good health remains a subject of debate. In recent years, however, emerging evidence highlights a U-shaped relationship between sleep duration and cardiovascular outcomes. While short sleep can contribute to cardiovascular stress through mechanisms like increased sympathetic activity and poor glucose regulation, long sleep durations may disrupt circadian rhythms and autonomic regulation, promoting inflammation and metabolic disturbances [2,3]. This emphasizes the importance of recognizing that both insufficient and excessive sleep may pose significant risks of increased risk of stroke, CAD, and total cardiovascular diseases (CVDs) such as hypertension and CAD [3,4]. 

Long sleep duration, typically defined as exceeding eight hours per night, has variously been categorized into thresholds such as >9 hours, >9 hours, and >10 hours [5-7]. Each of these thresholds represents increasingly extended sleep periods that are often considered in research studies. However, defining long sleep duration is further complicated by demographic factors significantly influencing sleep patterns. For instance, certain young individuals, certain cultural groups, and people with chronic health conditions tend to exhibit longer sleep durations [8,9]. Conversely, elderly adults, shift workers, and parents of young children frequently experience shorter sleep durations due to lifestyle demands and responsibilities [10].

Hypertension, CAD, and stroke are among the leading causes of global morbidity and mortality, collectively contributing to approximately 17.9 million deaths each year [11]. Hypertension alone affects over 33% of the global population, and stroke is a major contributor to disability-adjusted life years (DALYs) worldwide, with its prevalence particularly high in low- and middle-income countries [11]. Notably, sleep duration also plays a crucial role in cardiovascular health, impacting the development and progression of these conditions. Normal sleep patterns help regulate blood pressure, but deviations from typical sleep durations (too short or too long) can lead to adverse cardiovascular outcomes [12]. Hypertension, for instance, is often exacerbated by poor sleep quality and duration [13]. Similarly, both short and long sleep durations have been linked to an increased risk of CAD and stroke, highlighting the complex relationship between sleep and cardiovascular health [14]. Several established risk factors for CVD, such as genetic predisposition and lifestyle choices, intersect with sleep duration. Poor sleep habits can exacerbate other risk factors like obesity and diabetes, creating a compounding effect on cardiovascular risk [15].

Additionally, social factors such as race, socioeconomic status, and access to healthcare also play a crucial role in shaping cardiovascular outcomes. These determinants are intricately tied to disparities in sleep quality and duration, further compounding cardiovascular risk in vulnerable populations [16]. Understanding the nuanced impacts of both long and short sleep durations on cardiovascular health is crucial for developing effective interventions and promoting optimal sleep practices. This review examines the intricate relationship between sleep duration and cardiovascular health, providing insights into how different sleep patterns influence the risk and management of CVDs.

## Review

Methods

Search Strategy

A systematic search of electronic databases was conducted to identify relevant studies. The databases searched were PubMed/MEDLINE, Embase, and Web of Science. The “English” filter under Article Language was applied for all databases before the searches. The first search consisted of (“long sleep duration”) AND (“U-shape mechanism”) AND (“sleep duration”), whereas the second search consisted of (“long sleep duration”) AND (“cardiovascular diseases”) OR (“heart disease”). The third search consisted of (“long sleep duration”) AND (“hypertension”) OR (“coronary artery disease”) OR (“stroke”) OR (“heart failure”). The search strategy was adapted to suit each database's syntax and indexing conventions.

Study Selection

Two reviewers independently screened the titles and abstracts of identified articles to assess their eligibility for inclusion in the systematic review and imported them into Rayyan (Rayyan Systems Inc., Cambridge, MA) (https://www.rayyan.ai/). Rayyan is a web-based tool that facilitates systematic reviews by allowing collaborative screening, tagging, labeling, and filtering. It also includes a "blind mode" to prevent bias during independent screening by hiding the decisions of other reviewers. This web-based application has been used in other reviews before [17]. Table 1 summarizes the inclusion and exclusion criteria used to select studies. Studies were included if they involved adult populations and examined the relationship between sleep duration and cardiovascular health outcomes, focusing on long sleep duration. Exclusion criteria included non-peer-reviewed sources, studies on animal models, and studies focused solely on non-cardiovascular outcomes. Full-text articles of potentially eligible studies were retrieved and further assessed based on these predefined criteria. Any discrepancies between reviewers were resolved through consultation with a third reviewer.

**Table 1:** Inclusion and exclusion criteria applied during the literature search.

Inclusion criteria	Exclusion criteria
English text	Studies involving clinical data other than long sleep and cardiovascular health
Human studies	
Published from 2008 to 2024	
Gender: all	Study type: gray literature, conference abstracts, editorials, commentaries, and letters to the editor.
Age: all	
Any geographic location	
Study type: peer-reviewed systematic reviews, meta-analyses, cohort studies, case-control studies, cross-sectional studies, and randomized controlled trials.	

Data Extraction

Two reviewers independently extracted data using a standardized form. The data included study characteristics (e.g., author, year of publication, study design), participant characteristics (e.g., sample size, age, sex), conclusion (e.g., cardiovascular events, mortality), and key findings. Any discrepancies in the extracted data were resolved through discussion between reviewers.

Reporting

**Figure 1:**
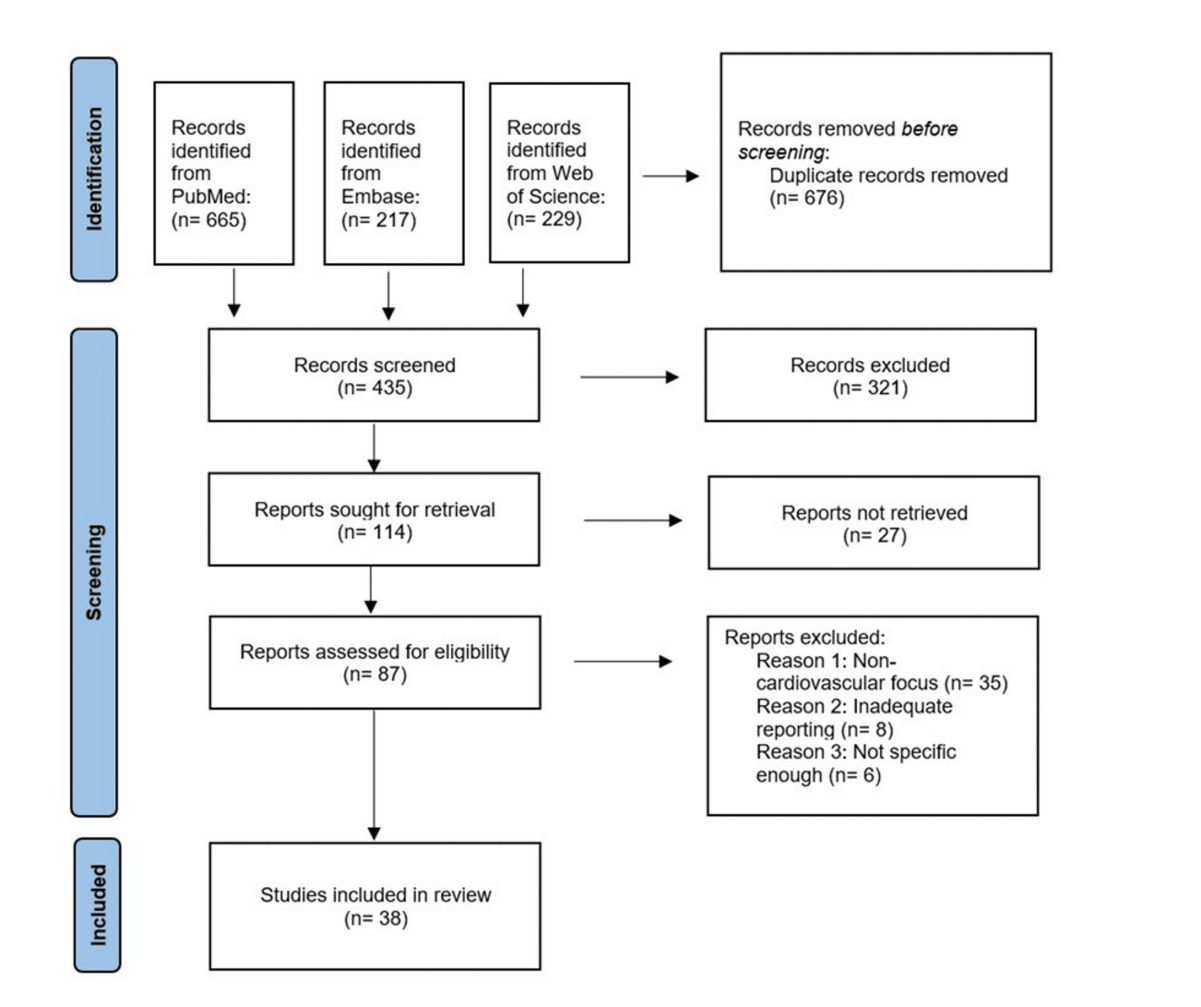
Flow diagram of the study selection process. Search results according to the Preferred Reporting Items for Systematic Reviews and Meta-Analyses (PRISMA).

The systematic review utilized the Preferred Reporting Items for Systematic Reviews and Meta-Analyses (PRISMA) 2020 guidelines, as shown in Figure 1 [18].

Results

As shown in the PRISMA diagram, during the initial search employing keywords and terms, we found 665 papers in PubMed, 217 in EMBASE, and 229 from Web of Science, yielding 1,111 articles. We removed 676 duplicate entries, and after further title and abstract screening, we eliminated 321 papers, leaving us with 114 articles. After a thorough review, 38 papers that addressed our research topic were left. Our final systematic review includes these 38 articles, as described in Table 2. 

**Table 2:** Summary of selected papers. This table summarizes studies that examined the relationship between sleep duration and cardiovascular health. The included studies are categorized by year, country, design, sample size, and population. The first column includes the paper's first author and the reference number. baPWV, brachial-ankle pulse wave velocity; BRFSS, Behavioral Risk Factor Surveillance System; CV, cardiovascular; HbA1c, glycated hemoglobin; HDL, high-density lipoprotein; IHD, ischemic heart disease; JACC, Japan Collaborative Cohort; KNHANES, Korea National Health and Nutrition Examination Survey; MACE, major adverse cardiovascular event; NCD, non-communicable disease; NHANES, National Health and Nutrition Examination Survey; NHIS, National Health Interview Survey; PSQI, Pittsburgh Sleep Quality Index; REGARDS, Reasons for Geographic and Racial Differences in Stroke; RR, relative risk

Study	Year	Country	Design	Sample size	Population	Duration	Outcome of interest	Findings
Garde et al. [19]	2013	Denmark	Prospective cohort study	5,249	Men aged 40-59 in Copenhagen Male Study	<6 hours (short) and ≥8 hours (long) vs. 6-7 hours	IHD mortality, all-cause mortality	Short sleep duration associated with increased IHD mortality in men.
Krittanawong et al. [20]	2020	USA	Cross-sectional analysis using NHANES data	32,152	NHANES participants, general US population	<7 hours (short) and >9 hours (long) vs. 7-9 hours (optimal)	Prevalence of coronary artery disease, heart failure, stroke, hypertension, diabetes, and hyperlipidemia	Long sleep (>9 hours) associated with stroke (OR 1.81) and heart failure (OR 1.47) compared to optimal sleep duration. Long durations linked to poor cardiovascular health outcomes.
Butler et al. [21]	2020	USA	Prospective cohort study	4,522	African American adults in the Jackson Heart Study	<6 hours (very short) and ≥9 hours (long) vs. 7-8 hours (recommended)	Incident coronary heart disease and stroke	Long and very short sleep duration linked to higher CVD hazard.
Lee et al. [22]	2015	South Korea	Cross-sectional study	29,203	Healthy young and middle-aged adults undergoing health screening	≤5, 6, 7, 8, ≥9 hours	Coronary artery calcification (CAC) and brachial-ankle pulse wave velocity (PWV)	U-shaped association between sleep duration and CAC/PWV; both short and long sleep linked to increased arterial stiffness.
Chen et al. [23]	2023	China	Prospective cohort study	409,156	Chinese adults without prior CVD or insomnia symptoms	≤5, 6, 7-8, 9, ≥10 hours	Stroke and coronary heart disease	Very short (≤5 hours) and very long (≥10 hours) sleep associated with increased risk of stroke and coronary heart disease.
Bochkarev et al. (The ESSE-RF Study) [24]	2019	Russia	Cross-sectional study	20,359	Adults aged 25-64 from 13 Russian regions	<6 hours (short) and >9 hours (long) vs. 7-8 hours	Hypertension, obesity, coronary artery disease, myocardial infarction	U-shaped association between sleep duration and coronary artery disease; J-shaped association with myocardial infarction.
Ikehara et al. (The JACC Study) [25]	2009	Japan	Prospective cohort study (JACC)	98,634	Japanese men and women aged 40-79	<5 hours (short) and ≥10 hours (long) vs. 7 hours	Mortality from stroke, coronary heart disease, all-cause mortality	Both short and long sleep duration associated with increased all-cause mortality and mortality from stroke and coronary heart disease.
Tsai et al. [26]	2014	Taiwan	Cross-sectional study	3,508	Adults aged 20-87 undergoing health examination in Taiwan	<6 hours (short), 6-8 hours (normal), >8 hours (long)	Increased arterial stiffness (measured by baPWV)	Long sleep duration associated with increased arterial stiffness in males; no association for short sleepers or females.
Lu et al. [27]	2020	China	Cross-sectional study using BRFSS data	1,191,768	General adult population in the US	≤5 hours (extremely short) and ≥10 hours (extremely long) vs. 7-8 hours (optimal)	Obesity, depression, diabetes, asthma, COPD, arthritis, kidney disease, CHD, stroke, cancer	Short and long sleep durations linked to higher risk of multiple chronic diseases in both genders such as CHD, stronger in ages 18-64.
Sands-Lincoln et al. [28]	2013	USA	Prospective cohort study	86,329	Postmenopausal women aged 50-79	≤5, 6, 7-8, ≥9 hours	CHD and CVD	Short and long sleep associated with higher CHD and CVD risk, with long sleep and high insomnia scores conferring the greatest risk.
Li et al. (The SAVE Study) [29]	2020	International	Multicenter prospective cohort study	2,687	Adults with obstructive sleep apnea and cardiovascular disease	<6, 6-8, >8 hours	Recurrent cardiovascular events, stroke	Long sleep (>8 hours) associated with increased risk of stroke but not other cardiac events.
Pan et al. [30]	2014	Singapore (Chinese population)	Prospective cohort study	63,257	Chinese adults aged 45-74	≤5, 6, 7, 8, ≥9 hours	Stroke mortality (ischemic and hemorrhagic)	Both short (≤5 hours) and long (≥9 hours) sleep linked to higher ischemic stroke mortality, particularly in those with hypertension.
Cheng et al. [31]	2022	United Kingdom	Prospective cohort study using UK Biobank	261,297	UK Biobank participants aged 39-64 years	<7 hours (short) and >9 hours (long) vs. 7-9 hours (referent)	Hypertension, stroke, CHD, DM, all-cause and cardiovascular mortality	Long sleep duration was independently associated with higher CMD risk and mortality.
Lalitnithi et al. [32]	2024	USA	Cross-sectional NHANES analysis	9,520	NHANES adult participants aged >18 years	<6, 6-9, >9 hours	Heart failure (HF) prevalence	Short (<6 hours) and long sleep (>9 hours) associated with increased HF risk (OR 1.70 and OR 1.53, respectively) compared to normal sleep (6-9 hours).
Lee et al. [33]	2018	Various	Case-control study	Not specified	Ischemic stroke survivors	>8 hours	Recurrent ischemic stroke	Long sleep duration associated with higher risk of stroke recurrence, particularly among those who previously experienced stroke events.
Kim et al. [34]	2016	Korea	Case-control study	1,470	Adults aged 30-84 from Korea	<6, 6-7, 8, and ≥9 hours	Intracerebral hemorrhage (ICH)	Long sleep (>8 hours) associated with increased ICH risk (OR 5.00 for ≥9 hours).
Liu et al. [35]	2022	China	Cross-sectional survey	5,065	Middle-aged and elderly in Guiyang	<7, 7-9, >9 hours	Stroke prevalence	Long sleep (>9 hours) associated with higher stroke prevalence; no association found for short sleep duration.
Wang et al. [36]	2020	Taiwan	Prospective cohort study	4,861	Healthy Taiwanese adults aged 20-68	<6 hours (short), 6-8 hours (reference), and >8 hours (long)	MACE, ischemic stroke, CV death	Long sleep associated with higher MACE risk (HR 1.317), ischemic stroke (HR 1.858) and CV events in adults.
Song et al. (The Kailuan Study) [37]	2016	China	Prospective cohort study	95,023	Chinese adults with no history of stroke at baseline	<6, 6-8, >8 hours	Ischemic and hemorrhagic stroke	Long sleep duration (>8 hours) associated with increased risk of total stroke (HR 1.29) and hemorrhagic stroke in women (HR 3.58).
Petrov et al. (The REGARDS Study) [38]	2018	USA	Prospective cohort study	16,733	Adults aged ≥45, Black and White, no history of stroke	<6, 6.0–6.9, 7.0–8.9, ≥9 hours	Incident stroke	Long sleep duration (≥9 hours) associated with higher stroke risk in White men; short sleep (<6 hours) associated with decreased stroke risk in Black men.
Titova et al. [39]	2020	Sweden	Prospective cohort study and Mendelian randomization	79,881	Adults aged 45-79 in Sweden	<7, 7-9, ≥9 hours	Total, ischemic, and hemorrhagic stroke	Long sleep (≥9 hours) linked with increased total and ischemic stroke risk, while short sleep (<7 hours) associated with higher risk of hemorrhagic stroke.
Fang et al. [40]	2014	USA	Cross-sectional analysis using NHIS	154,599	US adults aged 18 and older	≤6, 7-8, ≥9 hours	Stroke prevalence	Both short (≤6 hours) and long sleep (≥9 hours) linked with increased stroke prevalence; association varies by age and sex.
Kim et al. [41]	2018	Korea	Cross-sectional analysis using KNHANES data	17,601	Korean adults (≥19 years)	≤6 hours (short), 7-8 hours (normal), ≥9 hours (long)	Stroke prevalence	Long sleep (≥9 hours) associated with higher stroke prevalence, especially in women (OR 2.94, 95% CI 1.21 to 7.17). Short and long sleep linked to poor health outcomes.
Oikonomou et al. [42]	2021	Greece	Cross-sectional study	1,752	Middle-aged and older adults from the Corinthia region	<6, 6-7, 7-8, >8 hours	Carotid intima-media thickness (cIMT)	Very short and long sleep durations associated with increased cIMT, indicating greater atherosclerosis risk compared to normal sleep (7-8 hours).
Zhao et al. [43]	2020	China	Cross-sectional study	1,518	Health checkup population in China	<6, 6-7, 7-8, 8-9, >9 hours	Hypertension prevalence	Higher hypertension prevalence associated with short (6-7 hours) and long sleep (>9 hours), OR of 2.39 for long sleepers compared to 7-8 hours.
Chang et al. [44]	2022	China	Cross-sectional study using data from the China Kadoorie Biobank	55,687	Adults aged 30-79 from rural China	<6 hours, 6, 7, 8, and ≥9 hours	Hypertension prevalence	Long sleep (≥9 hours) associated with increased hypertension risk (OR 1.17); U-shaped relationship observed in females.
Budania et al. [45]	2023	India	Cross-sectional study	200	Patients aged 23-98 in North India	<6, 6-8, >8 hours	Hypertension	Long sleep (>8 hours) associated with increased hypertension risk; OR 1.301 (p < 0.01) compared with 6-8 hours.
Zhu et al. [46]	2023	China	Prospective cohort study	5,532	Pregnant women in early gestation	Sleep quality assessed with PSQI; <7, 7-8, ≥9 hours	Gestational hypertension and preeclampsia	Long sleep (≥9 hours) and poor sleep quality linked to increased risk of preeclampsia and gestational hypertension.
Huang et al. [47]	2021	China	Longitudinal study	3,178	Adults aged 30+ without hypertension at baseline	Persistent short (≤7 hours), normal (8-9 hours), long (≥10 hours)	Hypertension and inflammation	Persistent short and long sleep durations associated with higher risk of hypertension; short sleep associated with increased CRP levels.
Addo et al. [48]	2024	USA	Cross-sectional analysis using NHANES data	23,749	NHANES participants, general U.S. population	≤6 hours (short), 7-9 hours (recommended), ≥9 hours (long)	Prevalence of abnormal cardiovascular biomarkers (e.g., HDL, CRP, HbA1c, glucose)	Long sleep associated with abnormal CRP, HbA1c, and glucose.
Hale et al. [49]	2012	USA	Longitudinal study, Women’s Health Initiative	3,942	Postmenopausal women, WHI participants	5 hours or less, 6, 7-8, 9 or more hours	Incident coronary heart disease (CHD) and all-cause mortality	Long sleep duration (9+ hours) associated with increased odds of CHD and mortality, partially mediated by elevated fibrinogen levels.
Smiley et al. [50]	2019	USA	Cross-sectional analysis using NHANES data	2,705	NHANES 2013-2014 participants	≤5, 6, 7, 8, and ≥9 hours	HDL cholesterol, triglycerides, and LDL cholesterol	Long sleep duration non-linear associations observed for HDL and triglycerides. No significant association with LDL cholesterol.
Cui et al. [51]	2023	China	Prospective study	33,883	General population, aged 20-74	<7, 7-8, 8-9, >9 hours	Incident CVD	Long sleep (>9 hours) linked to higher CVD risk in participants aged ≥50; J-shaped relationship in older adults, with chronic health conditions contributing to risk.
Siengsukon et al. [52]	2020	USA	Perspective article	Not specified	General population with emphasis on diverse social determinants	Chronic sleep insufficiency and long sleep duration	Cardiovascular disease, obesity, type 2 diabetes mellitus, all-cause mortality	Long and short sleep durations are linked to increased cardiovascular and metabolic risks. Social determinants significantly influence sleep duration and quality, impacting CVD risks.
Ji et al. [53]	2020	China	Prospective cohort study	39,533	Adults in China, aged 18+	<6, 6-8, >8 hours	Stroke risk	Short and long sleep duration increased stroke risk; short duration with poor sleep quality posed the greatest risk with RR of 6.75.
Zhou et al. (The Dongfeng-Tongji Study) [54]	2024	China	Prospective cohort study	31,750	Older adults aged ~62 years at baseline in China	<6, 7-9, ≥9 hours	Incident stroke (total, ischemic, hemorrhagic)	Long sleep (≥9 hours) and poor sleep quality are associated with higher stroke risk; midday napping >90 min and long sleep increased total stroke risk by 85% (HR 1.85).
Im et al. [55]	2017	South Korea	Cross-sectional study	23,878	Korean adults aged 18+	<5, 6-8, >9 hours	Framingham risk score and CVD prevalence	Short and long sleep durations were both associated with higher CVD risk scores and prevalence of CVD.
Wu et al. [56]	2024	China	Prospective cohort study using NHANES	14,171	NHANES participants	<6 hours (short) and >8 hours (long) vs. 7-8 hours	All-cause mortality, heart disease mortality	Long sleep duration (>8 hours) was linked to higher mortality from all causes and heart disease in NCD patients.

**Figure 2:**
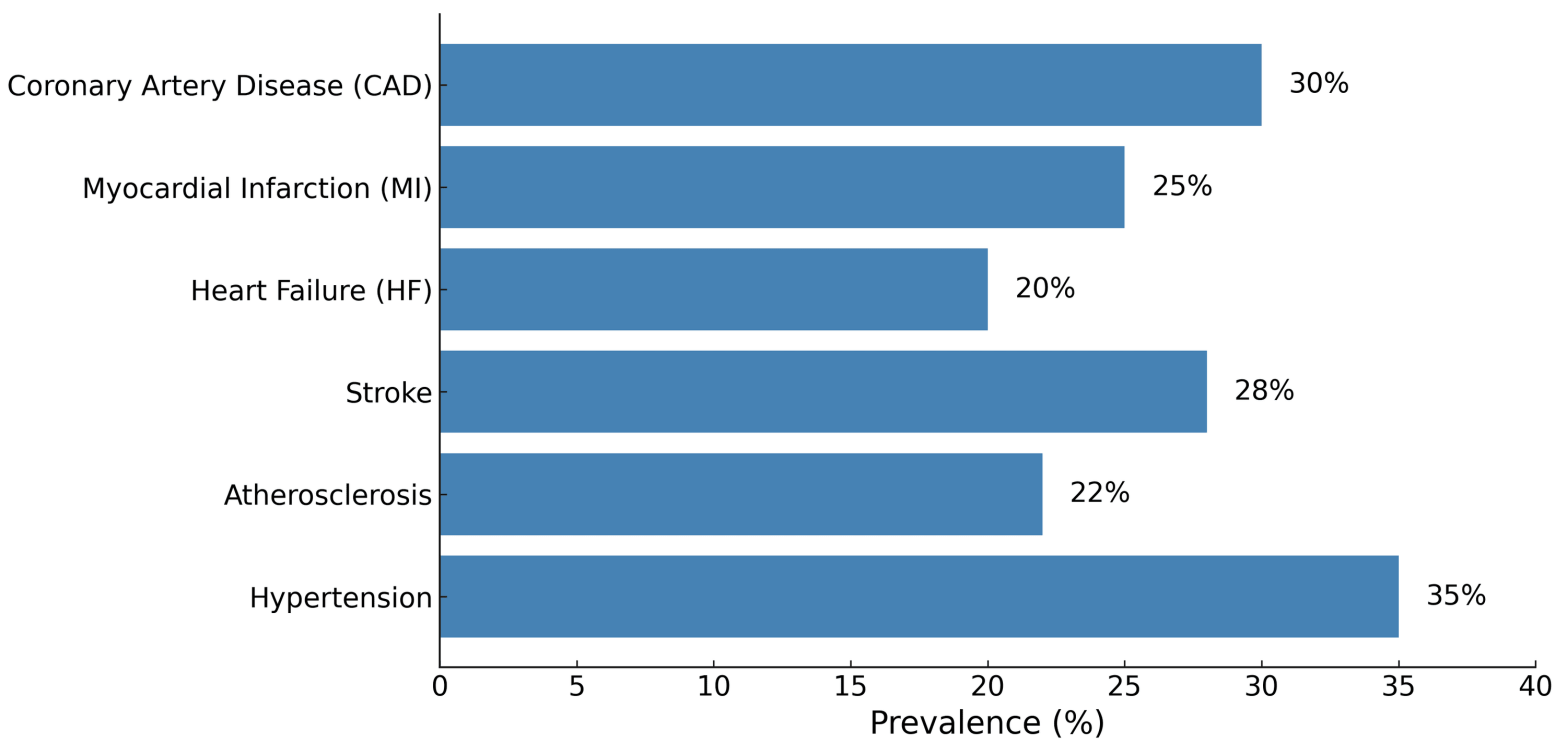
Prevalence of cardiovascular outcomes across studies. This figure, created by the authors, illustrates the prevalence of cardiovascular outcomes associated with long sleep duration across multiple studies.

The current systematic review evaluates the implications of long sleep duration on cardiovascular health, focusing on associations between long sleep and different cardiovascular outcomes such as CAD, myocardial infarction (MI), heart failure (HF), stroke, atherosclerosis, and hypertension. Based on the present findings, Figure 2 illustrates graphically the prevalence of cardiovascular complications linked to long sleep duration. Conditions such as hypertension (35%), CAD (30%), stroke (28%), MI (25%), atherosclerosis (22%), and HF (20%) show higher prevalence among individuals reporting long sleep durations (>9 hours per night).

The relation between sleep duration and cardiovascular outcomes based on this review’s findings is summarized in Table 3. The findings indicate a consistent link between prolonged sleep and adverse cardiovascular health, emphasizing that excessive sleep may not be as benign as commonly believed. This discussion will dive into the key cardiovascular conditions associated with long sleep, relevant inflammatory markers, and the potential influence of demographic and lifestyle factors, utilizing all reviewed studies to provide a comprehensive analysis.

**Table 3:** Summary of cardiovascular outcomes by sleep duration. This table summarizes the association between different sleep durations and various cardiovascular outcomes derived from the reviewed studies. Rows represent cardiovascular conditions, while columns categorize findings based on sleep duration: short (<6 hours), optimal (seven to eight hours), and long (>9 hours).

Outcome	<6 hours	7-8 hours	>9 hours
Stroke	Increased risk	Lower risk	Increased risk
Myocardial infarction	Increased risk	Lower risk	Increased risk
CAD	Increased risk	Lower risk	Increased risk
Hypertension	Increased risk	Lower risk	Increased risk
Heart failure	No significant finding	No significant finding	Increased risk
Atherosclerosis	Increased risk	Lower risk	No significant finding

Discussion

Long Sleep and CAD

Several studies suggest that long sleep duration is associated with an increased risk of CAD [19-25]. Individuals who sleep more than nine hours per night have significantly higher coronary artery calcium (CAC) scores, which indicate subclinical atherosclerosis, compared to those who sleep less than seven hours (short sleep) or seven to nine hours (optimal sleep) [20]. Specifically, the prevalence of CAD was highest in the group with long sleep duration (5.5% of 1,058 participants), compared to those with optimal sleep (3.7%) and short sleep (3.5%) [20]. Extended sleep has also been linked to elevated levels of inflammatory markers, such as CRP and fibrinogen, which contribute to the development and progression of CAD [57]. Elevated CRP levels have been associated with increased arterial stiffness, further exacerbating cardiovascular risk [22]. Brachial-ankle pulse wave velocity (baPWV), a measure of arterial stiffness, was significantly associated with long sleep duration, particularly in women and participants under the age of 60 [26]. Individuals with long sleep duration not only had higher CAC scores (>100 in 3.6% of participants) but also exhibited the highest baPWV values at 1,364.1 cm/second, highlighting an increased cardiovascular risk [22].

A U-shaped association between sleep duration and CAD risk has been consistently reported across multiple studies, with both extremely short (≤5 hours) and extremely long (>10 hours) sleep durations linked to higher rates of CAD [27]. Among these, those with extremely long sleep durations had the highest CAD prevalence at 8%, particularly among participants under the age of 65, suggesting that older age might be an important factor to consider in future research. The study by Bochkarev et al. [24] also confirmed this U-shaped relationship, reporting that long sleepers had an 11.9% prevalence of CAD compared to those with shorter or optimal sleep durations. This pattern of a U-shaped relationship has been observed in various populations, including postmenopausal women, adding further evidence that both insufficient and excessive sleep are associated with increased CAD risk [28]. Additionally, a population-based study by Lu et al. [27] found that long sleep duration was independently associated with CAD even after adjusting for traditional risk factors, such as hypertension, diabetes, and hyperlipidemia. These findings suggest that long sleep duration may be a modifiable risk factor for CAD and that interventions aimed at optimizing sleep duration could potentially reduce CAD risk.

Long Sleep and MI

The association between prolonged sleep and MI was consistently observed across multiple studies, with some reporting that individuals with long sleep duration (>8 hours) had a significantly higher incidence of MI compared to those with six to eight hours of sleep [29]. The underlying mechanisms may involve prolonged periods of inactivity, leading to endothelial dysfunction and reduced nitric oxide bioavailability, both of which are key factors in atherogenesis. Moreover, a study by Butler et al. [21] found that autonomic dysfunction, characterized by reduced heart rate variability and increased sympathetic activity, was prevalent among individuals with long sleep durations, increasing their susceptibility to ischemic events. The study by Pan et al. [30] further supported these findings, demonstrating that prolonged sleep was linked to increased arterial stiffness, a precursor to MI. The Sleep Apnea Cardiovascular Endpoints (SAVE) study by Li et al. [29] showed that long sleep duration was associated with a greater incidence of MI, even after adjusting for sleep-disordered breathing, suggesting that the relationship between long sleep and MI is independent of obstructive sleep apnea. A study using UK Biobank data by Cheng et al. [31] examined the relationship between excessive daytime sleepiness, which is known to be associated with CVD, and long sleep durations, which found a 91% increase in MI incidence. In comparison, long sleep without daytime sleepiness still showed a 39% increase in MI incidence. The researchers admit that the mechanisms behind the results are unclear and need more testing. These results highlight the importance of maintaining an optimal sleep duration to reduce the risk of MI, particularly in individuals with preexisting cardiovascular conditions.

Long Sleep and HF

Evidence from population-based studies supports the link between long sleep and HF. An analysis of the National Health and Nutrition Examination Survey (NHANES) data by Lalitnithi et al. [32] found that individuals with long sleep duration (>9 hours) had a higher prevalence of left ventricular hypertrophy and diastolic dysfunction, both of which are precursors to HF. Elevated CRP and fibrinogen levels, both markers of systemic inflammation, were consistently observed in individuals who slept excessively, suggesting a state of chronic inflammation that may contribute to myocardial remodeling and ventricular dysfunction [22]. The study by Butler et al. [21] also indicated that prolonged sleep duration was associated with increased sympathetic nervous system activity, which can lead to adverse cardiac remodeling. Previous longitudinal studies have found that individuals with long sleep had a higher risk of developing HF over a 10-year follow-up period, even after adjusting for confounding factors such as obesity, diabetes, and hypertension [58]. Mendelian randomization studies by Wang et al. have also identified a causal link between long sleep duration and increased risk of HF, further strengthening the evidence for this association [59].

Long Sleep and Stroke

The association between long sleep duration and stroke is well-documented, with prolonged sleep linked to an elevated risk of both ischemic and hemorrhagic stroke [33-41]. A nationwide case-control study by Kim et al. [34] found that individuals who slept more than nine hours per night had an increased risk of intracerebral hemorrhage. Similarly, it has been reported that long sleep duration was associated with a higher risk of ischemic stroke, particularly among older adults [35]. In a cohort of 98,634 subjects over the course of 13-15 years, there was a 1.5- to 2.5-fold increase in the risk of mortality from ischemic and total (of any nature) stroke in individuals sleeping nine hours or more per night [25]. The authors suggested that this increased risk might be explained by underlying health conditions, systemic inflammation, and impaired autonomic regulation associated with prolonged sleep duration. Another study noted that individuals sleeping longer than eight hours had a 1.8-fold increased risk of ischemic stroke after adjusting for age, sex, systolic blood pressure, diabetes, body mass index, low-density lipoprotein (LDL), and number of involved coronary arteries [36]. Mechanisms such as impaired cerebral autoregulation, systemic inflammation, and increased sympathetic nervous system activity were listed as possible reasons for the increased risk. These factors can all contribute to elevated stroke risk by impacting vascular health and increasing the likelihood of clot formation and other ischemic events. It has been reported that there is a significant increase in risk for hemorrhagic stroke among women, with long sleepers exhibiting upward of triple the probability of this cerebrovascular event [37]. Although the reasoning for this phenomenon is not fully known, it is hypothesized that this stark difference between values may be due to hormone levels and psychological factors between men and women. Many studies demonstrated associations between long sleep duration and stroke amongst specific populations. For example, a study by Petrov et al. [38] showed a strong association between White men who slept longer than nine hours and incidences of stroke. The study further mentions that this contrast has been shown in several meta-analyses; however, the reasoning for this is still unknown and shows some of the limits to the currently available literature. The SAVE study by Li et al. [29] showed that long-sleeping individuals with obstructive sleep apnea were at significantly higher risk for cerebrovascular events than those who were short-sleepers.

The three main explanations for these findings are that acute or long-term hypoxia will impact the brain differently from the heart in relation to variable collateral vasculature, the nocturnal biochemical and physiological disturbances such as acute post-apneic blood pressure surges, and the greater exposure to apnea events by sleeping for longer periods [29]. When examining the molecular mechanism involved, some studies further demonstrated that long sleep duration was associated with increased levels of inflammatory cytokines, including IL-6 and tumor necrosis factor-alpha (TNF-α), which contribute to the pathogenesis of stroke [21]. These findings underscore the importance of managing sleep duration in stroke survivors to reduce the risk of recurrence.

Long Sleep and Atherosclerosis

Long sleep has been implicated in the increased risk of atherosclerosis, potentially through both inflammatory and metabolic pathways. One study using carotid intima-media thickness (IMT) and CAC scoring demonstrated that individuals with longer sleep durations exhibited higher levels of subclinical atherosclerosis [22]. Chronic inflammation, as indicated by elevated CRP levels, appears to play a crucial role in mediating this relationship. It has been reported that long sleep was associated with increased levels of IL-6, which is another inflammatory marker implicated in atherosclerosis [60]. One predominant marker for CVD is increased IMT, which has been reported to be associated with long sleep duration [42,61]. Because increased IMT is a sign of subclinical atherosclerosis, it increases the risk for many forms of CVD. Carotid artery stiffness is less commonly utilized as a factor in directing clinical care but equally as effective in terms of predicting CVD [62]. A Mendelian randomization study demonstrated a potential causal relationship between long sleep and increased atherosclerotic burden [63]. Furthermore, it was reported that prolonged sleep was associated with increased arterial stiffness [20].

Long Sleep and Hypertension

Hypertension is another major cardiovascular condition linked to long sleep duration. The reviewed studies indicate that excessive sleep may disrupt normal circadian blood pressure regulation, leading to higher blood pressure and non-dipping patterns [20,24,30,31,43-47]. One study found that individuals with long sleep had a higher prevalence of non-dipping nocturnal blood pressure, potentially increasing the risk of left ventricular hypertrophy [20]. In a five-year study by Huang et al. [47], it was determined that long sleep duration leads to a significantly higher risk of developing hypertension. Another report by Budania et al. [45] found that the risk of hypertension was increased by 30.1% in participants with a long sleep duration (>8 hours/day) compared to those with normal (six to eight hours/day) sleep duration. A positive association between long sleep duration and hypertension has also been reported in subjects 45 years of age and older [43]. A prospective cohort study by Zhu et al. [46] examined 5,532 pregnant women in early gestation, the first 12 weeks of pregnancy, and compared their sleeping patterns to hypertensive risk and preeclampsia. The results of the study showed that long sleep duration (>9 hours/day) for pregnant females in early gestation was associated with a significant increase in blood pressure levels compared to those with normal sleep patterns, poor sleep quality, and even short sleep duration [53]. However, increased sleep duration was not associated with pre-eclampsia [46].

Inflammatory Markers

Inflammation appears to be a common pathway linking long sleep duration with adverse cardiovascular outcomes. Elevated CRP and fibrinogen levels have been consistently observed among individuals with prolonged sleep durations, indicating a state of chronic low-grade inflammation [47,64]. Previous studies have found that elevated CRP levels were associated with increased arterial stiffness and endothelial dysfunction, which are key factors in the development of CVDs [65]. In a five-year study., CRP levels were measured among individuals with varying sleep durations: short (<6 hours, N = 2,755), adequate (six to seven hours, N = 8,714), and long (>7 hours, N = 6,166) [66]. The study observed a U-shaped correlation between sleep duration and CRP levels, which was related to cardiovascular mortality. The unadjusted risk of CV mortality among those with short sleep and long sleep was 62% and 103% higher than those with optimal sleep. The elevated hazard remained significant after multivariate adjustment and was consistent among men and women [66].

A second study further examined the relationship between sleep duration and CRP, finding that long sleep duration was linked to increased odds of abnormal CRP levels and other biological markers such as high-density lipoprotein (HDL), glycated hemoglobin (HbA1c), and blood glucose [48]. This study categorized sleep durations as the following: short sleep (<6 hours) with 7,483 people, recommended duration (>6 hours to <9 hours) with 13,341 people, and long sleep (>9 hours) with 2,925 people. The long sleep category showed the highest abnormal levels of CRP (6.12 compared to 3.08 in short sleep), HbA1c (1.54 compared to 1.25 in short sleep), and glucose (1.45 compared to 1.09 in short sleep). 

Fibrinogen is a key inflammatory protein involved in blood clotting and serves as a valuable biomarker for assessing CVD risk [67]. Some studies have highlighted the correlation between long sleep duration and CVD, specifically measuring fibrinogen levels in women due to its association with increased odds of IMT more than 1.2 mm [49]. The study by Hale et al. [49] showed an association between long sleep (9+ hours) and fibrinogen levels and also reported increased odds of CHD compared to a control group of seven to eight hours of sleep. The strength of the association, as reported in the study, showed that long sleep duration was associated with an over twofold increase in the odds of CHD (OR = 2.05, 95% CI: 1.02-4.11) compared to the control group of seven to eight hours of sleep. This association suggests a notable increase in risk for CHD among those with extended sleep durations.

In the study of NHANES data from 2013-2014 by Smiley et al. [50], lipid panels were examined to understand the impact of sleep duration on lipid profiles in adults. Physicians often rely on these panels to assess the risk of CVD [68]. This study found that long sleep duration (>8 hours) was associated with adverse lipid levels, specifically higher serum triglycerides and lower HDL cholesterol levels in women. The highest mean HDL cholesterol was observed among participants who slept eight hours, while long sleepers exhibited lower mean HDL levels of 53.96 mg/dL and higher triglyceride levels of 127.36 mg/dL. Additionally, the analysis showed no significant association between sleep duration and LDL cholesterol across different sleep categories, with a mean LDL level of 108.55 mg/dL in the group sleeping over eight hours [50]. This data support the prevalent theory that long sleep duration is associated with obesity, metabolic syndrome, diabetes mellitus, and hypertension, which are all predisposing factors for CVD [69].

**Figure 3:**
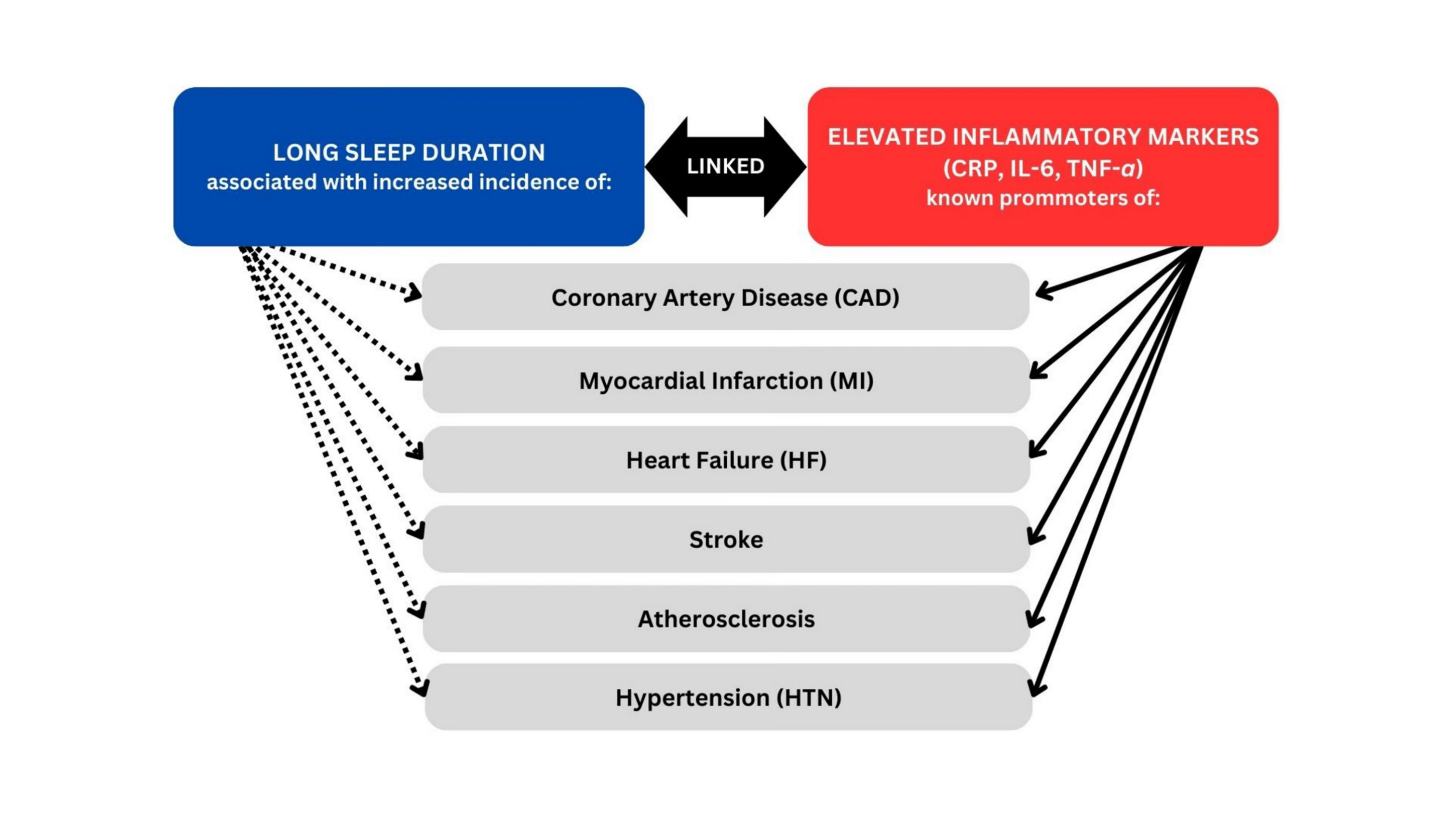
Inflammatory markers and cardiovascular risk. This figure, created by the authors, illustrates the role of inflammatory markers such as C-reactive protein (CRP), interleukin-6 (IL-6), and tumor necrosis factor-alpha (TNF-α) in mediating cardiovascular risks associated with sleep duration (>9 hours).

It has also been reported that long sleep was linked to increased levels of IL-6, further supporting the role of inflammation in mediating the relationship between extended sleep and cardiovascular risk [61,70]. TNF-α levels were also elevated in individuals with long sleep duration, suggesting a link between prolonged sleep and systemic inflammation [71]. Inflammatory markers are commonly associated with CVD risk, suggesting that a robust understanding of its interplay with sleeping patterns is crucial for developing effective CVD prevention and treatment strategies. A schema showing the various interactions is shown in Figure 3.

Demographic and Lifestyle Factors

The impact of long sleep duration on cardiovascular health is also influenced by demographic and lifestyle factors. Age, gender, socioeconomic status, and comorbid conditions are involved in determining the risk associated with prolonged sleep. For instance, older adults and individuals from lower socioeconomic backgrounds are more likely to report long sleep durations, partly due to comorbid conditions, poorer health status, and reduced access to healthcare [27,51]. One study by Butler et al. [21] found that African Americans with long sleep durations had a higher risk of CVD compared to their Caucasian counterparts. Lifestyle factors, including physical inactivity, obesity, and poor diet, are also more prevalent among those with prolonged sleep, compounding their cardiovascular risk [72]. The influence of sleep quality was also noted by several studies that reported that individuals with poor sleep quality and long sleep duration had a higher risk of cardiovascular events compared to those with good sleep quality [37,52-54]. For example, a Korean study by Im et al. [55] found that sleep durations over nine hours increased the risk for CVD based on the Framingham Risk Score, highlighting that insufficient and excessive sleep may elevate cardiovascular risks regardless of traditional risk factors. A different study found that individuals with lower educational attainment and lower socioeconomic status were more likely to experience long sleep durations [73]. Furthermore, individuals with sedentary lifestyles and poor dietary habits were more likely to have prolonged sleep, which further increased their risk of CVD [73,74].

Clinical Implications and Recommendations

The findings of this systematic review highlight the need for healthcare providers to recognize long sleep duration as a potential cardiovascular risk factor. Screening for excessive sleep and addressing underlying factors, such as obstructive sleep apnea, depression, or poor sleep quality, may help mitigate the associated cardiovascular risks [75]. Furthermore, promoting healthy sleep habits, including maintaining a regular sleep schedule and engaging in physical activity, may benefit cardiovascular health, particularly for individuals at high risk for CVDs [56]. Given the complex relationship between sleep and cardiovascular health, individualized interventions considering demographic, lifestyle, and comorbid factors are crucial for improving cardiovascular outcomes.

Limitations and Future Directions

Our systematic review has several limitations. First, including only English-language and peer-reviewed studies may have introduced selection bias, limiting the comprehensiveness of our findings. Second, the heterogeneity in defining long sleep duration across studies made it difficult to standardize the analysis, potentially affecting consistency. Third, due to variability in study designs and outcomes, we did not perform a meta-analysis, limiting our ability to quantify the overall effect of long sleep on cardiovascular outcomes.

While this review primarily focused on sleep duration, it is important to acknowledge the potential influence of sleep quality, particularly in populations with sleep disorders such as obstructive sleep apnea. Poor sleep quality often coexists with long sleep duration and may exacerbate cardiovascular risks through mechanisms such as inflammation and autonomic dysregulation. Future studies should investigate the combined impact of sleep quality and duration to provide a more nuanced understanding of their relationship with cardiovascular outcomes. Additionally, the bidirectional nature of the relationship between sleep and CVD warrants further exploration. While long sleep may increase the risk of cardiovascular conditions, existing CVD can, in turn, influence sleep patterns through factors such as fatigue, medication use, and reduced physical activity. Longitudinal studies are needed to assess these effects and clarify causal pathways.

## Conclusions

This systematic review highlights the significant association between long sleep duration and adverse cardiovascular outcomes, including CAD, MI, HF, stroke, atherosclerosis, and hypertension. While "too much" sleep is often defined as exceeding eight to nine hours per night, no universally accepted threshold has been established, reflecting variability in study methodologies and populations. These effects are likely mediated by mechanisms such as elevated inflammatory markers (e.g., CRP, IL-6, TNF-α), circadian rhythm disruption, and autonomic dysregulation. Clinicians should recognize long sleep duration as a potentially modifiable cardiovascular risk factor and incorporate sleep assessments into routine risk screening, particularly for older adults and individuals with comorbid conditions. This integration could improve prevention strategies and facilitate earlier interventions. Additionally, the findings underscore the need for future research to address key gaps, including the role of sleep quality, the bidirectional relationship between sleep and CVD, and disparities influenced by socioeconomic and demographic factors. These investigations could provide deeper insights into the physiological and social mechanisms driving these associations and inform targeted interventions. By emphasizing the clinical implications of long sleep duration and addressing its role in cardiovascular health, this review highlights the importance of integrating sleep assessments into public health and clinical care frameworks. Such efforts could mitigate cardiovascular risk and advance strategies for improving population health outcomes.
